# Myocardial Infarction and Traditional Cardiovascular Risk Factors in Older Patients in Primary Care

**DOI:** 10.1590/0034-7167-2024-0535

**Published:** 2025-11-28

**Authors:** Ellen Vanuza Martins Bertelli, Guilherme José Silva Ribeiro, Francisco Railson Bispo de Barros, Jacqueline Voltolini de Oliveira, Marcella Lima Marinho, André de Araújo Pinto

**Affiliations:** I Universidade Estadual de Roraima. Boa Vista, Roraima, Brazil; II Universidade Federal de Viçosa. Viçosa, Minas Gerais, Brazil

**Keywords:** Primary Health Care, Chronic Disease, Aged, Myocardial Infarction, Risk Factors., Atención Primaria de Salud, Enfermedad Crónica, Anciano, Infarto del Miocardio, Factores de Riesgo.

## Abstract

**Objectives:**

to investigate the prevalence of myocardial infarction and its association with cardiovascular risk factors in older adults in Primary Care.

**Methods:**

this cross-sectional study involves 1,322 older adults assisted in Basic Health Units in the northernmost part of Brazil. Data were collected in 2020, covering information on myocardial infarction, hypertension, diabetes, hypercholesterolemia, obesity, chronic kidney disease, and smoking.

**Results:**

myocardial infarction affected 10.5% of older adults, being more common in men (11.4% vs. 9.8%; p=0.327). Diabetes (OR=5.27; p<0.001), hypertension (OR=5.24; p<0.001), kidney disease (OR=5.20; p<0.001), and obesity (OR=2.87; p<0.001) significantly increased the odds of myocardial infarction.

**Conclusions:**

myocardial infarction affected 10% of older adults, and cardiovascular risk factors heightened this probability. Healthy lifestyle promotion and regular health monitoring are essential for its prevention.

## RESUMEN

**Objetivos:**

investigar la prevalencia de infarto de miocardio y su asociación con factores de riesgo cardiovascular en adultos mayores en Atención Primaria.

**Métodos:**

se trata de un estudio transversal con 1.322 adultos mayores atendidos en Unidades Básicas de Salud en el extremo norte de Brasil. Los datos fueron recolectados en 2020, abarcando información sobre infarto de miocardio, hipertensión, diabetes, hipercolesterolemia, obesidad, enfermedad renal crónica y tabaquismo.

**Resultados:**

el infarto de miocardio afectó al 10,5% de los adultos mayores, siendo más común en hombres (11,4% vs. 9,8%; p=0,327). La diabetes (OR=5,27; p<0,001), la hipertensión (OR=5,24; p<0,001), la enfermedad renal (OR=5,20; p<0,001) y la obesidad (OR=2,87; p<0,001) aumentaron significativamente las probabilidades de infarto de miocardio.

**Conclusiones:**

el infarto de miocardio afectó al 10% de los adultos mayores, y los factores de riesgo cardiovascular incrementaron esta probabilidad. La promoción de un estilo de vida saludable y el monitoreo regular de la salud son esenciales para su prevención.

## INTRODUCTION

Myocardial infarction (MI) is a serious condition caused by the sudden interruption of blood flow to the heart due to the obstruction of a coronary artery by a thrombus formed on an atherosclerotic plaque^(1)^. This condition represents a major and major public health concern worldwide, with cardiovascular disease leading to approximately 17.9 million deaths annually^(2)^. In Brazil, the rate of hospitalization for MI increased by 59.3% between 2011 and 2021^(3)^, and reached a staggering financial cost of R$22.4 billion (US$6.9 billion) in 2015, the most recent data available^(4)^.

With the global population aging, expectations regarding MI are concerning, as older adults face increased risk due to age-related changes and comorbidities^(5)^. Compared with younger patients, older adults with this condition often receive less invasive treatments and medications, which may reflect age-related bias and result in suboptimal care^(6)^. Furthermore, older adults also tend to accumulate more cardiovascular risk factors (CRFs) that increase the risk of MI, including hypertension^(7)^, diabetes^(8)^, chronic kidney disease (CKD)^(9)^, obesity^(10)^, high cholesterol levels^(11)^, and smoking^(12)^.

In this context, emerging CRFs, defined as recently identified biomarkers and conditions, including inflammation, oxidative stress, microRNAs, C-reactive protein, psychosocial factors and environmental pollutants, are also directly linked to MI^(13)^. However, traditional CRFs are well-established conditions that predispose to the development of MI, especially through atherosclerosis acceleration, and include hypercholesterolemia, obesity, diabetes, hypertension, and smoking^(14)^. The analysis of traditional CRFs allows a better understanding and management of the risk of MI, especially in contexts where resources for diagnosing new biomarkers are limited, such as Primary Health Care (PHC).

In Brazil, between 2006 and 2021, there was a significant increase in many of the traditional CRFs that overburden the Unified Health System (In Portuguese, *Sistema Único de Saúde* - SUS) and mainly affect older adults^(15)^. This trend is further aggravated by the country’s large regional disparities, with the northernmost part presenting more unfavorable socioeconomic conditions and less access to healthcare^(16)^. Regional differences, which also reflect variations in lifestyle, prevention plans and availability of medical diagnostic resources, also influence the prevalence of MI^(2)^.

MI represents an immense challenge for SUS, which offers free care to everyone, regardless of nationality, and is aggravated by socioeconomic and regional disparities in access to healthcare^(16)^. Additionally, PHC plays an important role in identifying and managing CRFs, which can improve MI prevention and treatment. Thus, considering the importance of PHC for public health and the increasing prevalence of CRFs^(15)^, conducting studies focused on MI in populations assisted by PHC is necessary.

Although MI and its determinants have been widely studied, gaps remain regarding its prevalence and association with traditional CRFs in the context of PHC in the northernmost part of Brazil. Regions with less access to specialized healthcare services may present distinct risk profiles and specific challenges for managing MI. Moreover, national data on the burden of MI in older adults treated in PHC are limited. This study, part of a broader investigation on older adults’ health in Roraima, aims to fill the gap in understanding the association between CRFs and MI. By obtaining epidemiological information, the findings will guide strategies for planning preventive and therapeutic actions, contributing to reducing the burden of MI in this vulnerable population.

## OBJECTIVES

To investigate the prevalence of MI and its association with traditional CRFs, including hypertension, diabetes, hypercholesterolemia, obesity, CKD, and smoking in older adults treated in PHC.

## METHODS

### Ethical aspects

The study was approved by the *Universidade Estadual de Roraima* Research Ethics Committee, in accordance with the Brazilian National Health Council (Resolution 466/12) and Declaration of Helsinki guidelines. Data were provided following confidentiality protocols, and written consent was obtained from health authorities.

### Study design, period and place

This observational cross-sectional study, based on the analysis of previously collected medical records, also presents characteristics of an ecological study, as it compares the occurrence of MI and its association with CRFs in population groups. The study was guided by the STrengthening the Reporting of OBservational studies in Epidemiology tool^(17)^, and is part of a larger study entitled “Cardiovascular risk factors and sequelae associated with SARS-CoV-2 virus infection in older adults in the state of Roraima”. The data are the responsibility of the Department of Epidemiological Surveillance (DES) of the State Department of Health of Roraima.

With 636,707 inhabitants and a total area of 223,644 km^2^, Roraima is the least populous state in Brazil, geographically located in the Amazon region, in the northernmost part of Brazil, with a population density of 2.85 people per km^2^. Only 15 municipalities make up the state of Roraima, most of which are centered in Boa Vista, the state capital that is home to 65% of the state’s population. With an average monthly household income of R$1,425 (about US$253), in 2023, the state ranked 15^th^ out of 27 Brazilian states in terms of income. The estimated life expectancy is 71.8 years, and Roraima’s Human Development Index is 0.699, showing advances in income and education. This information provides a context for examining the correlation between CRFs and MI.

### Study population, inclusion and exclusion criteria

According to the DES, 4,194 medical records were available for consultation, and a significant number of missing data in these records were observed. In view of this, it was necessary to establish a minimum sample estimate that would allow the exclusion of these incomplete records, eliminating the need for weighting methods^(18)^. This involved taking into account a two-design effect (deff) multiplier factor, a 95% Confidence Interval, an expected prevalence of 50% (for unknown outcomes), and an acceptable error of 4 percentage points. To account for losses, 20% was also added. These criteria indicated that at least 1,260 complete records were needed. Even with several missing data, in part because not all data were added in the health system, the final sample remained robust, totaling 1,322 older adults.

All records of older adults, defined as people aged 60 years or older, living in the state of Roraima and who received PHC services in 2020, were included. Inclusion was not limited by history of cardiovascular events, and older adults were included regardless of previous occurrence of MI, hypertension, diabetes, hypercholesterolemia, smoking, and CKD. Medical records that did not contain information on MI, hypertension, diabetes, hypercholesterolemia, smoking, CKD and essential sociodemographic data were excluded. However, all cases of MI were included, regardless of the presence or absence of specific CRFs.

### Study protocol

Data were collected by PHC healthcare professionals between January and December 2020. However, access to the records for this study was only granted in 2022, following institutional procedures for data release. Although the data were collected in 2020, they represent the most recent information available at the time of the request. The request involved data from all older adults treated in PHC during 2020, with the aim of contributing to broader research related to long COVID. In 2020, healthcare professionals, usually nurses and physicians, recorded the sociodemographic and health data of older adults on standardized forms, which were later entered into the DES system in binary format (yes or no), as established by PHC. Although registries contain diverse clinical and sociodemographic information of patients, this study focused exclusively on variables related to MI and its CRFs previously defined in the larger long COVID survey.

To describe MI (dependent variable) and other comorbidities, information was requested from the DES regarding the procedures adopted. According to the institution, the specialist physicians responsible for nosological diagnosis were, in large part, from specialized clinics that are partners. All venous blood samples were collected in partner laboratories or in the Basic Health Units themselves, with a recommended fasting period of at least 12 hours, and analyzed at the State Public Health Central Laboratory or partner institutions.

MI diagnosis was made following the Brazilian Society of Cardiology guidelines^(19)^. The initial screening considered the presence of angina, clinical history, physical examination and 12-lead electrocardiogram (ECG) performed within 10 minutes, in addition to troponin measurement. Possible alterations such as abnormal Q/QS waves and changes in ST-T segment were identified through ECG. After medical analysis, MI with ST-segment elevation or without ST-segment elevation was detected. In parallel, cardiac biomarkers such as troponins were measured, with elevated levels indicating damage to the heart muscle^(19)^. Finally, diagnosis was then confirmed and classified based on the combination of symptoms, ECG results and biomarkers. Only the presence or absence of MI was recorded in the DES information system, without distinction as to the type of elevation or infarct size. All individuals were included in the analysis regardless of these variables.

Specialist physicians from Basic Health Units or affiliated health units diagnosed traditional CRFs based on the Brazilian Ministry of Health guidelines. The Brazilian Society of Cardiology^(20)^, the Brazilian Society of Diabetes Mellitus^(21)^ and the Brazilian Guideline on Dyslipidemia and Prevention of Atherosclerosis^(22)^ were consulted in the development of diagnostic criteria and definitions. Hypertension diagnosis was made based on preserved blood pressure readings that were 140/90 mmHg or above^(20)^. A fasting blood glucose level of 126 mg/dL (7.0 mmol/L) or higher after an overnight fast and a hemoglobin A1c of at least 6.5% were used to diagnose diabetes^(21)^. According to laboratory diagnosis, hypercholesterolemia was classified as having low-density lipoprotein cholesterol blood levels of 130 mg/dL or higher^(22)^.

CKD was diagnosed by a specialist, following the Brazilian Society of Nephrology and Kidney Disease Outcomes Quality Initiative guidelines, based on assessment of glomerular filtration rate, proteinuria analysis and imaging tests. Diagnosis considered kidney damage lasting three months or more, indicated by structural or functional anomalies, with or without a decrease in glomerular filtration rate^(23)^. Smoking was defined as regular use of tobacco products according to National Cancer Institute guidelines. Smoking status was assessed by the question “Do you smoke?”, with the response options “Yes” or “No”.

Covariates that were recorded included age (measured in completed years), which was subsequently categorized into age group and sex (male or female). Moreover, skin color/race was defined based on self-identification following the national classification system. Thus, participants self-declared as belonging to one of the following categories: yellow, white, black, brown or indigenous. Data on place of residence (inland or capital) and educational level were also collected, with educational level classified as “no education”, “<8 years” or “≥8 years”, based on variations in responses.

### Analysis of results, and statistics

Data were analyzed using descriptive statistics, including distribution of absolute and relative frequencies, and inferential statistics. Student’s t-test was used to compare the age of older adults with and without MI, with normality of data confirmed by the Kolmogorov-Smirnov test. The chi-square test was used to explore possible interactions between MI and other variables. Significant differences in the prevalence of MI between older adults exposed and not exposed to CRFs were indicated by the non-overlapping 95% Confidence Intervals. Due to the binary nature of the data, unadjusted and adjusted binary logistic regressions were applied to investigate the associations between CRFs and MI. In multivariate analysis, all CRFs were adjusted for covariates, regardless of the p-value in the chi-square test. Analyses were conducted with IBM SPSS Statistics software (version 20.0; IBM Corp., Armonk, NY, USA), adopting a significance level of 5%.

## RESULTS

Table 1 describes the general characteristics of the 1,322 older patients, with a mean age of 70.4 (±7.87) years, treated in PHC. Participants were mainly women (55.0%), aged between 60 and 69 years (53.8%), brown skin color/race (45.2%), without formal education (47.2%) and residing in the state capital (64.1%). One in ten older adults (10.5%) was affected by MI, mainly those who were older (80 years or older) and without formal education. MI was slightly more frequent, but not statistically significant, among men (11.4%), indigenous people (15.7%) and residents of the capital (11.2%). Concerning comorbidities, while more than half of older adults had hypertension (75.3%), hypercholesterolemia (54.4%) and diabetes (51.9%), a smaller portion had CKD (16.8%), obesity (14.0%), and smoked (11.6%). All traditional CRFs were associated with MI (p<0.001), except smoking (p=0.372).

**Table 1:** General characteristics and prevalence of acute myocardial infarction in older patients, Roraima, Brazil (N=1,322)

Variables	General n (%)	Myocardial infarction		*p* value^*^
		Presence n (%)	95% CI	
	1322	139 (10.5)	8.9-11.9	^-^
**Sex**				
Female	727 (55.0)	71 (9.8)	8.2-11.1	0.327
Male	595 (45.0)	68 (11.4)	9.7-12.8	
**Age, mean (SD)**	70.4 (7.87)	74.7 (8.0)	73.2-76.1	<0.001^†^
**Age group**				
60-69	712 (53.8)	45 (6.3)	5.0-7.4	
70-79	416 (31.5)	44 (10.6)	9.0-12.0	<0.001
≥80	194 (14.7)	50 (25.8)	23.5-27.8	
**Skin color/race**				
Yellow	61 (4.6)	10 (11.5)	9.8-12.9	
White	302 (22.8)	29 (9.6)	8.0-10.9	
Black	222 (16.8)	23 (10.4)	8.8-11.8	0.165
Brown	597 (45.2)	58 (9.7)	8.2-11.0	
Indigenous	140 (10.6)	22 (15.7)	13.9-17.3	
**Education**				
No education	624 (47.2)	94 (15.1)	13.2-16.7	
< 8 years	395 (29.9)	25 (6.3)	5.0-7.4	<0.001
≥ 8 years	303 (22.9)	20 (6.6)	5.3-7.7	
**Place of residence**				
Countryside	475 (35.9)	44 (9.3)	7.8-10.6	0.267
Capital	847 (64.1)	95 (11.2)	9.5-12.6	
**Hypercholesterolemia**				
Yes	719 (54.4)	107 (14.9)	13.0-16.5	<0.001
No	603 (45.6)	32 (5.3)	4.2-6.3	
**Hypertension**				
Yes	995 (75.3)	132 (13.3)	11.5-14.8	<0.001
No	327 (24.7)	10 (3.0)	2.1-3.8	
**Diabetes**				
Yes	686 (51.9)	118 (17.2)	15.2-18.8	<0.001
No	636 (48.1)	21 (3.3)	2.4-4.1	
**Obesity**				
Yes	185 (14.0)	35 (18.9)	16.9-20.7	<0.001
No	1137 (86.0)	104 (9.1)	7.6-10.4	
**Kidney disease**				
Yes	222 (16.8)	66 (29.7)	27.2-31.7	<0.001
No	1100 (83.2)	73 (6.6)	5.3-7.7	
**Smoking**				
Yes	154 (11.6)	126 (10.8)	9.2-12.2	0.372
No	1168 (88.4)	13 (8.4)	7.0-9.6	

n - relative frequency; % - absolute frequency; SD - standard deviation;

* Chi-square test; †Independent t-test.

**Figure 1:**
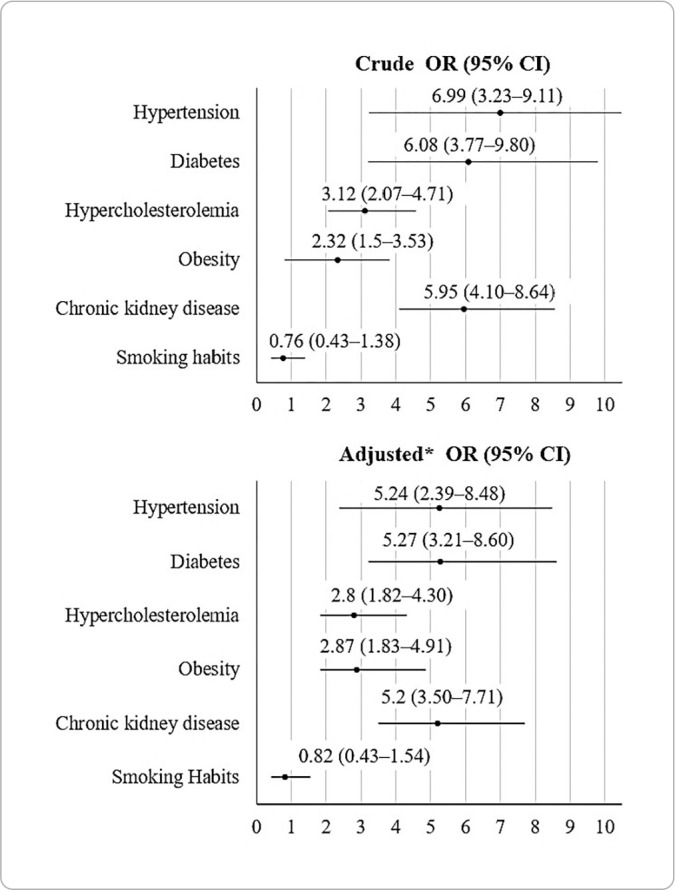
(Unadjusted and adjusted) logistic regression of cardiovascular risk factors associated with myocardial infarction in older adults

Figure 1 presents the unadjusted and adjusted analyses of binary logistic regression for the association of traditional CRFs with MI. In unadjusted analysis, with the exception of smoking (OR=0.76; 95% CI=0.42-1.28), the presence of any of CRFs increased the odds of MI in older adults treated in PHC. In analysis adjusted for all covariates, the associations remained the same. Compared to their peers without the presence of the respective comorbidities, older adults with hypercholesterolemia (OR=2.80; 95% CI=1.82-4.30), hypertension (OR=5.24; 95% CI=2.39-8.48) and diabetes (OR=5.27; 95% CI=3.21-8.60) had significantly increased odds of having MI. This same association was also observed when comparing obese versus non-obese individuals (OR=2.87; 95% CI=1.83-4.91) and those with kidney disease versus those without kidney disease (OR=5.20; 95% CI=3.50-7.71). Smoking was not associated with MI among study participants (p=0.529).

*Adjusted for all covariates.

## DISCUSSION

This study contributes to the understanding of MI in older adults treated in the context of PHC. The prevalence of 10% found is higher than the global estimate for this age group, reinforcing the need for specific preventive and care strategies for this population. Furthermore, the analysis of the association between CRFs and MI in this context fills an important gap, since most studies focus on hospitals and specialized services, while PHC represents the main gateway to the Brazilian health system. Adjusted analysis showed that hypertension, diabetes, hypercholesterolemia, obesity and CKD increase the chances of infarction. These findings have important implications. First, they reinforce the need for prevention and control of the investigated CRFs, and the need for continuous monitoring. Second, the results encourage the development of actions such as education, counseling, rehabilitation programs and integrated care to improve the quality of life of older adults affected by MI.

Epidemiological information on MI in Brazil is not widely available. This important health problem that affects more than 10.5% of our older population may be even more widespread in a country with continental dimensions such as Brazil. With more than 200 million inhabitants distributed across five large regions with distinct environmental, cultural and economic characteristics, it is important to understand the extent of this problem throughout the country. Globally, the prevalence of MI discovered by a meta-analysis was estimated at 3.8% in individuals under 60 years of age and 9.5% in those over 60 years of age^(2)^. Although the numbers found here exceed the global estimate, MI may be more common in patients with specific clinical conditions. For instance, a study conducted with more than 7,545 patients revealed that the occurrence of MI can vary significantly between 3.6% and 59.6% in patients with hypertensive emergencies^(7)^. Furthermore, many older patients may have experienced unrecognized MI, due to the absence of overt symptoms or lack of awareness of the event. This is supported by studies showing that between 22% and 64% of patients experience unrecognized MI, with atypical symptoms or no MI symptoms^(24,25)^.

Hypertension increased the chances of older adults having MI by more than five times. In fact, in outpatients, hypertension is considered one of the main determinants of cardiovascular events in older adults, including MI^(1,7,26)^. However, the magnitude of the association found in this study is higher than that reported in international cohorts^(27)^, which may be related to poor blood pressure control in Brazilian PHC patients. In older hypertensive patients, uncontrolled high blood pressure levels are even more worrying because they increase the risk of heart failure and mortality^(27,28)^, which reinforces the need for more effective interventions at this level of care. This relationship between MI and hypertension probably occurs because hypertension damages the vascular endothelium, promoting the formation of thrombi (thrombogenesis) that can obstruct the coronary arteries and lead to MI^(7)^. Hypertension also activates the renin-angiotensin-aldosterone system, elevating levels of angiotensin II, which promotes vasoconstriction, inflammation, and vascular remodeling, while angiotensin II and aldosterone contribute to cardiac and vascular fibrosis, increasing the risk of MI^(29)^. Another possible explanation for this relationship is related to the failure of vascular autoregulation in hypertension, which increases peripheral vascular resistance, causing damage to the heart and potentially leading to MI^(7)^. Thus, strict control of blood pressure in older adults is imperative, not only to prevent hypertension but also to mitigate the risks of MI and other cardiovascular diseases.

The results also revealed that older adults with hypercholesterolemia and obesity were more likely to have an MI. Previous studies have shown that hypercholesterolemia^(11,30)^ and obesity^(10,31)^ significantly increase the risk of MI. High cholesterol levels contribute to the formation of atherosclerotic plaques^(32)^, which in turn favors the progression of ischemic heart disease and reduced capacity to solubilize additional cholesterol^(33)^. The direct relationship between hypercholesterolemia and MI is conceived because high cholesterol directly affects the myocardium, altering the expression of cytoskeletal and contractile proteins, which can lead to cardiac dysfunction and, consequently, the development of MI^(34)^. Although the association between hypercholesterolemia and MI is well established, the present study expands this evidence by examining this relationship in a PHC setting. Thus, the results highlight the importance of PHC in prevention, with systematic screening and early interventions for cholesterol control in at-risk populations, aiming to reduce the incidence of cardiovascular events.

In the case of obesity, its impact on MI is multifaceted and presents a paradox: while obesity increases the risk of MI, it may also have protective effects^(10)^. Metabolically unhealthy obesity phenotypes are associated with a higher risk of MI and worse outcomes^(35)^. Additionally, the relationship between obesity and infarct size varies by type of MI. For instance, obesity is associated with larger infarcts in non-ST-segment elevation myocardial infarction and smaller infarcts in ST-segment elevation myocardial infarction^(10)^. Therefore, although the relationship between obesity and MI remains controversial, its association with patient outcomes highlights the need for further research to elucidate the pathophysiological mechanisms involved.

Older adults with CKD had increased odds of MI, supporting other studies^(9,36)^. Patients with CKD often have less access to invasive treatment and coronary revascularization, which contributes to higher in-hospital mortality compared to patients without CKD^(37)^. However, when patients with CKD receive invasive treatment for MI, in-hospital mortality is lower compared with conservative treatment, regardless of CKD severity^(37)^. Two main processes may explain this association, whose pathophysiology involves two overlapping processes: atherosclerosis and arteriosclerosis, the latter being predominant^(38)^. CKD causes chronic inflammation, which leads to changes in blood vessels and the heart, including atherosclerotic lesions, vascular calcification, and myocardial fibrosis^(39)^. These changes increase arterial stiffness and pressure, increasing the risk of arrhythmias, heart failure, and MI^(38)^. As kidney function worsens, the risk of severe MI and in-hospital mortality increases^(40)^. Despite this evidence, there are gaps in Brazilian literature regarding the existence and assessment of the effectiveness of cardiovascular prevention strategies in older adults with CKD in PHC, where early management can prevent complications and hospitalizations. In practical terms, nurses working in PHC can contribute to a more accurate screening of CRFs that affect renal function in older adults, promoting preventive interventions before progression to serious cardiovascular events.

In our study, the odds of MI were five times higher in older adults with diabetes. Diabetes has been reported as a critical risk factor for MI in studies conducted in Bangladesh^(41)^, the United States^(8)^, and other countries^(2)^. Diabetes can lead to MI for a variety of reasons, including metabolic problems, oxidative stress, and inflammation^(42)^. Moreover, chronic hyperglycemia causes excess glucose in heart cells, which leads to oxidative stress and accumulation of advanced glycation end products^(43)^. This results in mitochondrial dysfunction, impaired calcium signaling, and activation of multiple signaling pathways, culminating in changes in cardiac muscle and impaired heart function^(42,44)^. Furthermore, diabetes increases the risk of kidney damage after MI due to activation of the toll-like receptor and reactive oxygen species generated by NASDH oxidase^(45)^. Thus, the implementation of early screening and intensive management strategies in primary care may be crucial to reducing the impact of diabetes on the occurrence of MI and its associated complications.

### Study limitations

Study limitations need to be considered. First, the study’s cross-sectional design does not allow us to establish causal relationships between comorbidities and MI, even though several studies corroborate our findings^(7-11)^. Second, since the data were provided by the DES, and we were not responsible for entering this information into the system, there is a possibility of underreporting^(18)^. Furthermore, since many participants were excluded due to lack of complete information, the comprehensiveness and accuracy of our conclusions may have been affected. Third, since tobacco consumption data were self-reported, we cannot rule out that the lack of association between smoking and MI may have occurred due to the low number of positive reports for smoking. This may be common, since older patients may provide socially desirable responses due to the sensitivity of the topic. Fourth, we do not rule out important losses due to the use of dichotomized data that do not predict the severity or intensity of the medical condition and statistical sensitivity. Fifth, the data are limited to a specific region of Brazil, a country of continental dimensions, where different regions present distinct climatic, cultural and behavioral characteristics^(18)^. Consequently, these findings may not be applicable to older adults in other regions. Finally, the lack of information such as income and alcohol consumption may influence the relationship between CRFs and MI, constituting a significant limitation.

### Contributions to nursing and health

The results of the study highlight the role of nursing in PHC, especially in identifying older adults with MI and in managing CRFs. Nursing professionals can be strong allies in strategies involving health education, continuous monitoring, blood pressure control, and therapeutic adherence. In addition, nurses can help promote self-care among patients, guiding adherence to prescribed therapies. The study emphasizes early diagnosis and proactive management of chronic diseases in PHC, areas in which nursing can implement integrated strategies to reduce cardiovascular complications and improve the quality of life of older adults. Thus, the study strengthens nursing practice, highlighting the need for coordinated and individualized actions in the management of patients at risk of cardiac events.

## CONCLUSIONS

One in ten older adults treated in PHC had MI, which is more frequent among those with hypertension, diabetes, hypercholesterolemia, obesity and CKD, revealing the need for more effective prevention and management strategies for these CRFs. Addressing this challenge involves developing integrated health education and guidance programs, promoting cardiovascular rehabilitation and ensuring continuous monitoring of older adults’ health conditions. It is imperative that future research explore the prevalence and determinants of MI in other regions of Brazil, considering the country’s epidemiological and sociodemographic diversity.

## Data Availability

The research data are available only upon request.
