# Supplementary material for: Myocardial Infarction and Traditional Cardiovascular Risk Factors in Older Patients in Primary Care
Source: Rev Bras Enferm. 2025 Nov 28;78(5):e20240535. doi: 10.1590/0034-7167-2024-0535 (PMC12673614; doi:10.1590/0034-7167-2024-0535)
Supplement: Translation

# Translation: Infarto do Miocárdio e Fatores de Risco Cardiovascular Tradicionais em Idosos na Atenção Primária

**DOI:** 10.1590/0034-7167-2024-0535pt

**Authors:** Ellen Vanuza Martins Bertelli, Guilherme José Silva Ribeiro, Francisco Railson Bispo de Barros, Jacqueline Voltolini de Oliveira, Marcella Lima Marinho, André de Araújo Pinto

**Affiliations:** I Universidade Estadual de Roraima. Boa Vista, Roraima, Brasil; II Universidade Federal de Viçosa. Viçosa, Minas Gerais, Brasil

**Keywords:** Atenção Primária à, Saúde, Doença Crônica, Idoso, Infarto do Miocárdio, Fatores de Risco.

## Abstract

**Objetivos:**

investigar a prevalência de infarto do miocárdio e sua associação com fatores de risco cardiovascular em idosos na Atenção Primária.

**Métodos:**

trata-se de estudo transversal com 1.322 idosos atendidos em Unidades Básicas de Saúde no extremo norte brasileiro. Os dados foram coletados em 2020, abordando informações sobre infarto do miocárdio, hipertensão, diabetes, hipercolesterolemia, obesidade, doença renal crônica e tabagismo.

**Resultados:**

o infarto do miocárdio afetou 10,5% dos idosos, sendo mais comum em homens (11,4% vs. 9,8%; p=0,327). Diabetes (OR=5,27; p<0,001), hipertensão (OR=5,24; p<0,001), doença renal (OR=5,20; p<0,001) e obesidade (OR=2,87; p<0,001) aumentaram significativamente as chances de infarto do miocárdio.

**Conclusões:**

o infarto do miocárdio afetou 10% dos idosos, e os fatores de risco cardiovascular aumentaram essa probabilidade. A promoção de um estilo de vida saudável e o monitoramento regular da saúde são essenciais para sua prevenção.

## INTRODUÇÃO

O infarto do miocárdio (IM) é uma condição grave causada pela interrupção súbita do fluxo sanguíneo para o coração devido à obstrução de uma artéria coronária por um trombo formado sobre uma placa aterosclerótica^(1)^. Essa condição representa um problema importante e de grande impacto para a saúde pública em nível mundial, com as doenças cardiovasculares levando a aproximadamente 17,9 milhões de mortes anualmente^(2)^. No Brasil, a taxa de hospitalização por IM aumentou 59,3% entre 2011 e 2021^(3)^, e alcançou um custo financeiro impressionante de R$ 22,4 bilhões (6,9 bilhões de dólares) em 2015, o dado mais recente disponível^(4)^.

Com a população global envelhecendo, as expectativas em relação ao IM são preocupantes, pois os idosos enfrentam maior risco devido a alterações e comorbidades relacionadas à idade^(5)^. Comparados com pacientes mais jovens, os idosos com essa condição frequentemente recebem tratamentos e medicamentos menos invasivos, o que pode refletir um viés relacionado à idade e resultar em cuidados subótimos^(6)^. Ademais, os idosos também tendem a acumular mais fatores de risco cardiovascular (FRCs) que aumentam o risco de IM, incluindo hipertensão^(7)^, diabetes^(8)^, doença renal crônica (DRC)^(9)^, obesidade^(10)^, níveis elevados de colesterol^(11)^ e tabagismo^(12)^.

Nesse contexto, os FRCs emergentes, definidos como biomarcadores e condições recentemente identificadas, incluindo inflamação, estresse oxidativo, microRNAs, proteína C reativa, fatores psicossociais e poluentes ambientais também estão diretamente ligados ao IM^(13)^. Não obstante, os FRCs tradicionais são condições bem estabelecidas que predispõem ao desenvolvimento de IM, especialmente por meio da aceleração da aterosclerose, e incluem hipercolesterolemia, obesidade, diabetes, hipertensão e tabagismo^(14)^. A análise dos FRCs tradicionais permite uma melhor compreensão e gestão do risco de IM, especialmente em contextos onde os recursos para diagnóstico de novos biomarcadores são limitados, como a Atenção Primária à Saúde (APS).

No Brasil, entre 2006 e 2021, houve um aumento expressivo de muitos dos FRCs tradicionais que sobrecarregam o Sistema Único de Saúde (SUS) e afetam principalmente os idosos^(15)^. Essa tendência é ainda agravada pelas grandes disparidades regionais do país, com o extremo norte apresentando condições socioeconômicas mais desfavoráveis e menor acesso à saúde^(16)^. As diferenças regionais, que também refletem variações no estilo de vida, nos planos de prevenção e na disponibilidade de recursos de diagnóstico médico, também influenciam a prevalência de IM^(2)^.

O IM representa um imenso desafio para o SUS, que oferece atendimento gratuito a todos, independentemente da nacionalidade, sendo agravado por disparidades socioeconômicas e regionais no acesso à saúde^(16)^. Adicionalmente, a APS ocupa um papel importante na identificação e manejo dos FRCs, podendo melhorar a prevenção e o tratamento do IM. Assim, considerando a importância da APS para a saúde pública e a crescente prevalência dos FRCs^(15)^, a condução de estudos focados no IM em populações atendidas na APS se faz necessária.

Embora o IM e seus determinantes sejam amplamente estudados, ainda persistem lacunas quanto à sua prevalência e associação com os FRCs tradicionais no contexto da APS no extremo norte do Brasil. Regiões com menor acesso a serviços especializados de saúde podem apresentar perfis distintos de risco e desafios específicos para o manejo do IM. Além disso, dados nacionais sobre a carga do IM em idosos atendidos na APS são limitados. Este estudo, inserido em investigação mais ampla sobre a saúde da população idosa em Roraima, visa preencher a lacuna existente na compreensão da associação entre os FRCs e IM. Por meio da obtenção de informações epidemiológicas, os achados orientarão estratégias para o planejamento de ações preventivas e terapêuticas, contribuindo para a redução da carga do IM nessa população vulnerável.

## OBJETIVOS

Investigar a prevalência de IM e sua associação com FRCs tradicionais, incluindo hipertensão, diabetes, hipercolesterolemia, obesidade, DRC e tabagismo em idosos atendidos na APS.

## MÉTODOS

### Aspectos éticos

O estudo foi aprovado pelo Comitê de Ética em Pesquisa da Universidade Estadual de Roraima, conforme as diretrizes do Conselho Nacional de Saúde (Resolução n^o^ 466/12) e da Declaração de Helsinque. Os dados foram fornecidos seguindo protocolos de confidencialidade, sendo obtido consentimento das autoridades de saúde por escrito.

### Desenho, período e local do estudo

Este estudo transversal observacional, baseado na análise de registros médicos previamente coletados, apresenta também características de estudo ecológico, pois compara a ocorrência do IM e sua associação com FRCs em agregados populacionais. O estudo foi norteado pela ferramenta *STrengthening the Reporting of OBservational studies in Epidemiology* ^(17)^, e faz parte de pesquisa maior intitulada “Fatores de risco cardiovascular e sequelas associadas à infecção pelo vírus Sars-COV-2 em idosos do estado de Roraima”. Os dados são de competência do Departamento de Vigilância Epidemiológica (DVE) da Secretaria de Estado da Saúde de Roraima.

Com 636.707 habitantes e uma área total de 223.644 km^2^, Roraima é o estado menos populoso do Brasil, geograficamente localizado na região amazônica, no extremo norte do país, possuindo densidade populacional de 2,85 pessoas por km^2^. Apenas 15 municípios compõem o estado de Roraima, a maioria dos quais está centrada em Boa Vista, a capital do estado que abriga 65% da população do estado. Com uma renda familiar média mensal de R$ 1.425, ou cerca de 253 dólares, em 2023, o estado ficou em 15º lugar entre 27 estados brasileiros em termos de renda. A expectativa de vida estimada é de 71,8 anos, e o Índice de Desenvolvimento Humano de Roraima é de 0,699, mostrando avanços em renda e educação. Essas informações oferecem um contexto para examinar a correlação entre os FRCs e IM.

### População do estudo, critérios de inclusão e exclusão

Conforme o DVE, 4.194 registros médicos se encontravam disponíveis para consulta, e um número expressivo de dados ausentes nesses registros foi observado. Diante disso, foi necessário estabelecer uma estimativa de amostra mínima que permitisse a exclusão desses registros incompletos, eliminando a necessidade de métodos de ponderação^(18)^. Isso envolveu levar em consideração um fator multiplicador de dois efeitos de *design (deff)*, um Intervalo de Confiança de 95%, uma prevalência esperada de 50% (para resultados desconhecidos) e um erro aceitável de 4 pontos percentuais. Para compensar perdas, 20% também foram adicionados. Esses critérios indicaram que, no mínimo, 1.260 registros completos eram necessários. Mesmo com vários dados ausentes, em parte porque nem todos os dados foram adicionados no sistema de saúde, a amostra final permaneceu robusta, totalizando 1.322 idosos.

Foram incluídos todos os registros dos idosos, definidos como pessoas com 60 anos ou mais, residentes no estado de Roraima e que receberam serviços de saúde primários no ano de 2020. A inclusão não foi limitada por histórico de eventos cardiovasculares, e idosos foram incluídos independentemente da ocorrência prévia de IM, hipertensão, diabetes, hipercolesterolemia, tabagismo e DRC. Foram excluídos os registros médicos que não continham informações sobre o IM, hipertensão, diabetes, hipercolesterolemia, tabagismo, DRC e dados sociodemográficos essenciais. No entanto, todos os casos de IM foram incluídos, independentemente da presença ou ausência de FRCs específicos.

### Protocolo do estudo

Os dados foram coletados por profissionais de saúde da APS entre janeiro e dezembro de 2020. No entanto, o acesso aos registros para realização deste estudo foi concedido apenas em 2022, seguindo os trâmites institucionais para liberação de dados. Apesar de serem dados coletados em 2020, eles representam a informação mais recente disponível no momento da solicitação. A solicitação envolveu os dados de todas as pessoas idosas atendidas na APS durante o ano de 2020, com o objetivo de contribuir para pesquisa mais ampla relacionada à COVID longa. Em 2020, os profissionais de saúde, geralmente enfermeiros e médicos, registraram os dados sociodemográficos e de saúde dos idosos em formulários padronizados, posteriormente inseridos no sistema DVE em formato binário (sim ou não), conforme estabelecidos pela APS. Embora os registros contenham diversas informações clínicas e sociodemográficas dos pacientes, este estudo focou exclusivamente nas variáveis relacionadas ao IM e seus FRCs previamente definidos na pesquisa maior sobre COVID longa.

Para a descrição do IM (variável dependente) e das demais comorbidades, solicitaram-se informações do DVE quanto aos procedimentos adotados. De acordo com a instituição, os médicos especialistas responsáveis pelo diagnóstico nosológico eram, em grande parte, de clínicas especializadas conveniadas. Todas as amostras de sangue venoso foram coletadas em laboratórios conveniados ou nas próprias Unidades Básicas de Saúde, com jejum recomendado de pelo menos 12 h, e analisadas no Laboratório Central de Saúde Pública do Estado ou instituições conveniadas.

O diagnóstico de IM foi realizado seguindo as Diretrizes Brasileiras de Cardiologia^(19)^. A triagem inicial considerou a presença de angina, o histórico clínico, exame físico e eletrocardiograma (ECG) de 12 derivações realizado em até 10 minutos, além da aferição de troponina. As possíveis alterações como ondas Q/QS anormais e alterações no segmento ST-T foram identificadas por meio do ECG. Após análise médica, foi detectado IM com supradesnivelamento do segmento ST ou sem supradesnivelamento do segmento ST. Em paralelo, foram medidos os biomarcadores cardíacos, como troponinas, com níveis elevados indicando dano ao músculo cardíaco^(19)^. Finalmente, o diagnóstico foi então confirmado e classificado com base na combinação dos sintomas, resultados do ECG e biomarcadores. Apenas a presença ou ausência de IM foi registrada no sistema de informações do DVE, sem distinção quanto ao tipo de supradesnivelamento ou tamanho do infarto. Todos os indivíduos foram incluídos na análise independentemente dessas variáveis.

Médicos especialistas das Unidades Básicas de Saúde ou conveniados diagnosticaram os FRCs tradicionais com base nas diretrizes do Ministério da Saúde do Brasil. A Sociedade Brasileira de Cardiologia^(20)^, a Sociedade Brasileira de Diabetes Mellitus^(21)^ e a Diretriz Brasileira de Dislipidemias e Prevenção da Aterosclerose^(22)^ foram consultadas no desenvolvimento dos critérios e definições diagnósticas. O diagnóstico de hipertensão foi feito com base na preservação de leituras de pressão arterial que eram 140/90 mmHg ou acima^(20)^. Um nível de glicemia de jejum de 126 mg/dL (7,0 mmol/L) ou superior após um jejum noturno e uma hemoglobina A1c de pelo menos 6,5% foram usados para diagnosticar diabetes^(21)^. De acordo com o diagnóstico laboratorial, a hipercolesterolemia foi classificada como tendo níveis sanguíneos de lipoproteína de baixa densidade de 130 mg/dL ou mais^(22)^.

A cronicidade da DRC foi diagnosticada por um especialista, seguindo as diretrizes da Sociedade Brasileira de Nefrologia e do *Kidney Disease Outcomes Quality Initiative*, com base na avaliação da taxa de filtração glomerular, análise de proteinúria e exames de imagem. O diagnóstico considerou lesão renal com duração de três meses ou mais, indicada por anomalias estruturais ou funcionais, com ou sem diminuição da taxa de filtração glomerular^(23)^. O tabagismo foi considerado como uso regular de produtos de tabaco, de acordo com as diretrizes do Instituto Nacional do Câncer. O *status* de tabagismo foi avaliado pela pergunta “Você fuma?”, com as opções de resposta “Sim” ou “Não”.

As covariáveis que foram registradas incluíram idade (medida em anos completos), que foi posteriormente categorizada em faixa etária e sexo (masculino ou feminino). Além disso, a cor da pele/raça foi definida a partir da autoidentificação seguindo o sistema de classificação nacional. Dessa forma, os participantes se autodeclaram como pertencentes a uma das seguintes categorias: amarelo, branco, preto, pardo ou indígena. Dados sobre o local de moradia (interior ou capital) e o nível educacional também foram coletados, com o nível de escolaridade classificado como “sem educação”, “<8 anos” ou “≥8 anos”, com base nas variações nas respostas.

### Análise dos resultados e estatística

Os dados foram analisados utilizando estatística descritiva, incluindo distribuição de frequências absolutas e relativas, e estatística inferencial. O teste t de Student foi empregado para comparar a idade dos idosos com e sem IM, com a normalidade dos dados confirmada pelo teste de Kolmogorov-Smirnov. O teste qui-quadrado foi utilizado para explorar as possíveis interações entre o IM e outras variáveis. Diferenças significativas nas prevalências de IM entre os idosos expostos e não expostos a FRCs foram indicadas pela não sobreposição dos Intervalos de Confiança de 95%. Devido à natureza binária dos dados, foram aplicadas regressões logísticas binária bruta e ajustada para investigar as associações entre os FRCs e IM. Na análise multivariada, todos os FRCs foram ajustados pelas covariáveis, independentemente do valor de p no teste qui-quadrado. As análises foram conduzidas com o *software IBM SPSS Statistics* (versão 20.0; IBM Corp., Armonk, NY, EUA), adotando um nível de significância de 5%.

## RESULTADOS

As características gerais dos 1.322 pacientes idosos, com média de idade de 70.4 (±7.87) anos, atendidos na APS estão descritas na Tabela 1. Os participantes eram principalmente mulheres (55,0%), com idade entre 60 e 69 anos (53,8%), da cor da pele/raça parda (45,2%), sem estudo formal (47,2%) e residindo na capital do estado (64,1%). Um em cada dez idosos (10,5%) foi acometido por IM, principalmente os mais velhos (80 anos ou mais) e sem estudo formal. O IM foi ligeiramente mais frequente, mas não estatisticamente significativo, entre os homens (11,4%), indígenas (15,7%) e residentes na capital (11,2%). Quanto às comorbidades, enquanto mais da metade dos idosos tinha hipertensão (75,3%), hipercolesterolemia (54,4%) e diabetes (51,9%), uma parcela menor tinha DCR (16,8%), obesidade (14,0%) e o hábito de fumar (11,6%). Todos os FRCs tradicionais se associaram ao IM (*p<*0,001), exceto o tabagismo (*p*=0,372).

**Tabela 1:** Características gerais e prevalência de infarto agudo do miocárdio em idosos, Roraima, Brasil (N=1.322)

Variáveis	Geral n (%)	Infarto do miocárdio		valor de *p* ^*^
		Presença n (%)	IC 95%	
	1322	139 (10,5)	8,9-11,9	^-^
**Sexo**				
Mulher	727 (55,0)	71 (9,8)	8,2-11,1	0,327
Homem	595 (45,0)	68 (11,4)	9,7-12,8	
**Idade, média (DP)**	70,4 (7,87)	74,7 (8,0)	73,2-76,1	<0,001^†^
**Faixa etária**				
60-69	712 (53,8)	45 (6,3)	5,0-7,4	
70-79	416 (31,5)	44 (10,6)	9,0-12,0	<0,001
≥80	194 (14,7)	50 (25,8)	23,5-27,8	
**Cor da pele/raça**				
Amarela	61 (4,6)	10 (11,5)	9,8-12,9	
Branca	302 (22,8)	29 (9,6)	8,0-10,9	
Preta	222 (16,8)	23 (10,4)	8,8-11,8	0,165
Parda	597 (45,2)	58 (9,7)	8,2-11,0	
Indígena	140 (10,6)	22 (15,7)	13,9-17,3	
**Escolaridade**				
Sem estudo	624 (47,2)	94 (15,1)	13,2-16,7	
< 8 anos	395 (29,9)	25 (6,3)	5,0-7,4	<0,001
≥ 8 anos	303 (22,9)	20 (6,6)	5,3-7,7	
**Local de residência**				
Interior	475 (35,9)	44 (9,3)	7,8-10,6	0,267
Capital	847 (64,1)	95 (11,2)	9,5-12,6	
**Hipercolesterolemia**				
Sim	719 (54,4)	107 (14,9)	13,0-16,5	<0,001
Não	603 (45,6)	32 (5,3)	4,2-6,3	
**Hipertensão**				
Sim	995 (75,3)	132 (13,3)	11,5-14,8	<0,001
Não	327 (24,7)	10 (3,0)	2,1-3,8	
**Diabetes**				
Sim	686 (51,9)	118 (17,2)	15,2-18,8	<0,001
Não	636 (48,1)	21 (3,3)	2,4-4,1	
**Obesidade**				
Sim	185 (14,0)	35 (18,9)	16,9-20,7	<0,001
Não	1137 (86,0)	104 (9,1)	7,6-10,4	
**Doença renal**				
Sim	222 (16,8)	66 (29,7)	27,2-31,7	<0,001
Não	1100 (83,2)	73 (6,6)	5,3-7,7	
**Tabagismo**				
Sim	154 (11,6)	126 (10,8)	9,2-12,2	0,372
Não	1168 (88,4)	13 (8,4)	7,0-9,6	

n - frequência relativa; % - frequência absoluta; SD - desvio padrão;

* Teste qui-quadrado;

† Teste t independente.

As análises bruta e ajustada da regressão logística binária para a associação dos FRCs tradicionais com o IM estão apresentadas na Figura 1. Na análise bruta, com exceção do tabagismo (OR=0,76; IC 95%=0,42-1,28), a presença de qualquer um dos FRCs aumentou as chances de IM nos idosos atendidos na APS. Na análise ajustada por todas as covariáveis, as associações permaneceram as mesmas. Comparados aos seus pares sem a presença das respectivas comorbidades, os idosos com hipercolesterolemia (OR=2,80; IC 95%=1,82-4,30), hipertensão (OR=5,24; IC 95%=2,39-8,48) e diabetes (OR=5,27; IC 95%=3,21-8,60) tiveram chances significativamente aumentadas de terem IM. Essa mesma associação também foi observada comparando obesos *versus* não obesos (OR=2,87; IC 95%=1,83-4,91) e com doença renal *versus* sem doença renal (OR=5,20; IC 95%=3,50-7,71). O hábito de fumar não se associou ao IM entre os participantes do estudo (p=0,529).

**Figura 1:** Regressão logística (bruta e ajustada) de fatores de risco cardiovascular associados ao infarto do miocárdio em adultos mais velhos

*Ajustada para todas as covariáveis.

## DISCUSSÃO

Este estudo contribui para o entendimento do IM na população idosa atendida no contexto da APS. A prevalência de 10,5% encontrada é superior à estimativa global para essa faixa etária, reforçando a necessidade de estratégias preventivas e assistenciais específicas para essa população. Além disso, a análise da associação entre os FRCs e IM nesse contexto preenche uma lacuna importante, visto que a maioria dos estudos foca em hospitais e serviços especializados, enquanto a APS representa a principal porta de entrada do sistema de saúde brasileiro. A análise ajustada mostrou que hipertensão, diabetes, hipercolesterolemia, obesidade e DRC aumentam as chances de infarto. Esses achados têm implicações importantes. Em primeiro lugar, eles reforçam a necessidade de prevenção e controle dos FRCs investigados, e a necessidade de monitoramento contínuo. Em segundo lugar, os resultados suscitam o desenvolvimento de ações como educação, aconselhamento, programas de reabilitação e atenção integrada para melhorar a qualidade de vida dos idosos afetados pelo IM.

Informações epidemiológicas sobre IM no Brasil não estão amplamente disponíveis. Esse importante problema de saúde que afetou mais de 10,5% dos nossos idosos pode ser ainda mais abrangente em um país de dimensões continentais como o Brasil. Com mais de 200 milhões de habitantes distribuídos em cinco grandes regiões com características ambientais, culturais e econômicas distintas, é importante entender a extensão desse problema em todo o território nacional. Globalmente, a prevalência de IM descoberta por uma meta-análise foi estimada em 3,8%, em indivíduos com menos de 60 anos, e 9,5%, em maiores de 60 anos^(2)^. Embora os números aqui encontrados superem a estimativa global, o IM pode ser mais comum em pacientes com condições clínicas especificas. Por exemplo, estudo conduzido com mais de 7.545 pacientes revelou que a ocorrência de IM pode variar significativamente entre 3,6% e 59,6% em pacientes com emergências hipertensivas^(7)^. Além disso, muitos pacientes idosos podem ter experimentado IM sem diagnóstico, devido à ausência de sintomas evidentes ou falta de consciência do evento. Isso é corroborado por estudos que mostram que entre 22% e 64% dos pacientes experimentam IM não reconhecido, com sintomas atípicos ou sem sintomas de IM^(24,25)^.

A hipertensão aumentou em mais de cinco vezes as chances de idosos terem IM. Na verdade, em pacientes ambulatoriais, a hipertensão é considerada um dos principais determinantes de eventos cardiovasculares em idosos, incluindo o IM^(1,7,26)^. No entanto, a magnitude da associação encontrada neste estudo é superior à relatada em coortes internacionais^(27)^, o que pode estar relacionado ao baixo controle da pressão arterial em pacientes da APS brasileira. Em pacientes idosos hipertensos, níveis pressóricos elevados não controlados são ainda mais preocupantes porque aumentam o risco de insuficiência cardíaca e mortalidade^(27,28)^, o que reforça a necessidade de intervenções mais eficazes nesse nível de atenção. Essa relação entre o IM e hipertensão provavelmente ocorre porque a hipertensão danifica o endotélio vascular, promovendo a formação de trombos (trombogênese) que podem obstruir as artérias coronárias e levar ao IM^(7)^. A hipertensão também ativa o sistema renina-angiotensina-aldosterona, elevando os níveis de angiotensina II, que promove vasoconstrição, inflamação e remodelação vascular, enquanto a angiotensina II e aldosterona contribuem para a fibrose cardíaca e vascular, aumentando o risco de IM^(29)^. Outra possível explicação para essa relação está relacionada à falha na autorregulação vascular na hipertensão, que aumenta a resistência vascular periférica, causando danos ao coração e potencialmente levando ao IM^(7)^. Assim, é imperativo o controle rigoroso da pressão arterial em idosos, não apenas para prevenir a hipertensão, mas também para mitigar os riscos de IM e outras doenças cardiovasculares.

Os resultados revelaram ainda que os idosos com hipercolesterolemia e obesidade tinham maiores chances de ter um IM. Estudos prévios atestaram que a hipercolesterolemia^(11,30)^ e obesidade^(10,31)^ aumentam significativamente o aumento do risco de IM. Níveis elevados de colesterol contribuem para a formação de placas ateroscleróticas^(32)^, o que, por sua vez, favorece a progressão das doenças isquêmicas do coração e pela capacidade reduzida de solubilizar o colesterol adicional^(33)^. A relação direta entre hipercolesterolemia e IM é concebida porque o colesterol alto afeta diretamente o miocárdio, alterando a expressão de proteínas citoesqueléticas e contráteis, o que pode levar à disfunção cardíaca e, consequentemente, ao desenvolvimento do IM^(34)^. Embora a associação entre hipercolesterolemia e IM esteja bem estabelecida, o presente estudo amplia essa evidência ao examinar essa relação em um cenário de APS. Assim, os resultados destacam a importância da APS na prevenção, com rastreamento sistemático e intervenções precoces para controle do colesterol em populações de risco, visando reduzir a incidência de eventos cardiovasculares.

No caso da obesidade, seu impacto no IM é multifacetado e apresenta um paradoxo: enquanto a obesidade aumenta o risco de IM, também pode ter efeitos protetores^(10)^. Fenótipos de obesidade metabolicamente não saudáveis estão associados a um maior risco de IM e a piores desfechos^(35)^. Adicionalmente, a relação entre obesidade e tamanho do infarto varia conforme o tipo de IM. Por exemplo, a obesidade está associada a um infarto maior no infarto agudo do miocárdio (IAM) sem supradesnivelamento do segmento ST e a infartos menores no IAM com supradesnivelamento^(10)^. Portanto, embora a relação entre a obesidade e IM permaneça controversa, sua associação com os desfechos dos pacientes ressalta a necessidade de mais pesquisas para elucidar os mecanismos fisiopatológicos envolvidos.

Os idosos com DRC apresentaram chances aumentadas de IM, corroborando outros estudos^(9,36)^. Pacientes com DRC frequentemente têm menos acesso a tratamento invasivo e revascularização coronária, o que contribui para uma maior mortalidade hospitalar em comparação com pacientes sem DRC^(37)^. No entanto, quando pacientes com DRC recebem tratamento invasivo para o IM, a mortalidade hospitalar é menor em comparação ao tratamento conservador, independentemente da gravidade da DRC^(37)^. Dois processos principais podem explicar essa associação, cuja fisiopatologia envolve dois processos sobrepostos: aterosclerose e arteriosclerose, sendo esta última predominante^(38)^. A DRC causa inflamação crônica, que leva a alterações nos vasos sanguíneos e no coração, incluindo lesões ateroscleróticas, calcificação vascular e fibrose miocárdica^(39)^. Essas mudanças aumentam a rigidez arterial e a pressão, elevando o risco de arritmias, insuficiência cardíaca e IM^(38)^. À medida que a função renal piora, o risco de IM grave e mortalidade hospitalar aumenta^(40)^. Apesar dessas evidências, há lacunas na literatura brasileira quanto à existência e avaliação da efetividade das estratégias de prevenção cardiovascular em idosos com DRC no contexto da APS, onde o manejo precoce pode evitar complicações e hospitalizações. Em termos práticos, enfermeiros que atuam na APS podem contribuir para um rastreamento mais preciso dos FRCs que afetam a função renal dos idosos, promovendo intervenções preventivas antes da progressão para eventos cardiovasculares graves.

Em nosso estudo, as chances de IM foram cinco vezes maiores nos idosos com diabetes. O diabetes foi relatado como fator de risco crítico para o IM em estudo realizados em Bangladesh^(41)^, Estados Unidos^(8)^ e em outros países^(2)^. O diabetes pode levar ao IM por diversos motivos, incluindo problemas no metabolismo, estresse oxidativo e inflamação^(42)^. Além disso, a hiperglicemia crônica causa excesso de glicose nas células do coração, o que leva ao estresse oxidativo e acúmulo de produtos finais de glicação avançada^(43)^. Isso resulta em disfunção das mitocôndrias, problemas na sinalização de cálcio e ativação de várias vias de sinalização, culminando em alterações no músculo cardíaco e problemas na função do coração^(42,44)^. Ademais, o diabetes aumenta o risco de danos nos rins após um IM, devido à ativação do receptor *toll-like* e das espécies reativas de oxigênio geradas pela NADPH oxidase^(45)^. Dessa forma, a implementação de estratégias de rastreamento precoce e manejo intensivo na APS pode ser determinante para reduzir o impacto do diabetes na ocorrência de IM e suas complicações associadas.

### Limitações do estudo

As limitações do estudo precisam ser consideradas. Em primeiro lugar, o desenho transversal do estudo não permite estabelecer relações causais entre as comorbidades e IM, mesmo que diversos estudos corroborem nossos achados^(7-11)^. Em segundo lugar, como os dados foram fornecidos pelo DVE, e não fomos responsáveis pela inserção dessas informações no sistema, há uma possibilidade de subnotificação^(18)^. Além disso, como muitos participantes foram excluídos devido à ausência de informações completas, a abrangência e precisão das nossas conclusões podem ter sido afetadas. Em terceiro lugar, como os dados do consumo de tabaco foram autorrelatados, não descartamos que a não associação do tabagismo como o IM pode ter ocorrido pela baixa quantidade de relatos positivos para o tabagismo. Isso pode ser comum, visto que os pacientes idosos podem fornecer respostas socialmente desejáveis, devido à sensibilidade do tema. Em quarto lugar, não descartamos perdas importantes devido ao uso de dados dicotomizados que não predizem a gravidade ou intensidade da condição médica e a sensibilidade estatística. Em quinto lugar, os dados são limitados a uma região específica do Brasil, um país de dimensões continentais, onde diferentes regiões apresentam características climáticas, culturais e comportamentais distintas^(18)^. Consequentemente, esses achados podem não ser aplicáveis a idosos em outras regiões. Finalmente, a ausência de informações como renda e consumo de álcool pode influenciar a relação entre os FRCs e IM, constituindo uma limitação significativa.

### Contribuições para as áreas da enfermagem e saúde

Os resultados do estudo ressaltam o papel da enfermagem na APS, sobretudo na identificação de idosos com IM e na gestão de FRCs. Os profissionais de enfermagem podem ser fortes aliados em estratégias envolvendo educação em saúde, monitoramento contínuo, controle da pressão arterial e adesão terapêutica. Além disso, enfermeiros podem ajudar na promoção do autocuidado entre os pacientes, orientando a adesão às terapias prescritas. O estudo enfatiza o diagnóstico precoce e a gestão proativa de doenças crônicas na APS, áreas em que a enfermagem pode implementar estratégias integradas para reduzir complicações cardiovasculares e melhorar a qualidade de vida dos idosos. Assim, o estudo fortalece a prática de enfermagem, destacando a necessidade de ações coordenadas e individualizadas no manejo de pacientes em risco de eventos cardíacos.

## CONCLUSÕES

Um em cada dez idosos atendidos na APS tiveram IM, sendo mais frequente entre aqueles com hipertensão, diabetes, hipercolesterolemia, obesidade e DRC, revelando a necessidade de estratégias de prevenção e manejo mais eficazes para esses FRCs. O enfretamento desse desafio passa pelo desenvolvimento de programas integrados de educação em saúde e orientação, promovendo a reabilitação cardiovascular e garantindo um monitoramento contínuo das condições de saúde dos idosos. É imperativo que pesquisas futuras explorem a prevalência e os determinantes do IM em outras regiões do Brasil, considerando a diversidade epidemiológica e sociodemográfica do país.

## Data Availability

Os dados de pesquisa só estão disponíveis mediante solicitação.
